# SynLight: a bicistronic strategy for simultaneous active zone and cell labeling in the *Drosophila* nervous system

**DOI:** 10.1093/g3journal/jkad221

**Published:** 2023-09-27

**Authors:** Michael A Aimino, Jesse Humenik, Michael J Parisi, Juan Carlos Duhart, Timothy J Mosca

**Affiliations:** Department of Neuroscience, Vickie and Jack Farber Institute of Neuroscience, Thomas Jefferson University, Bluemle Life Sciences Building, Philadelphia, PA 19107, USA; Department of Neuroscience, Vickie and Jack Farber Institute of Neuroscience, Thomas Jefferson University, Bluemle Life Sciences Building, Philadelphia, PA 19107, USA; Department of Neuroscience, Vickie and Jack Farber Institute of Neuroscience, Thomas Jefferson University, Bluemle Life Sciences Building, Philadelphia, PA 19107, USA; Department of Neuroscience, Vickie and Jack Farber Institute of Neuroscience, Thomas Jefferson University, Bluemle Life Sciences Building, Philadelphia, PA 19107, USA; Department of Neuroscience, Vickie and Jack Farber Institute of Neuroscience, Thomas Jefferson University, Bluemle Life Sciences Building, Philadelphia, PA 19107, USA

**Keywords:** synapse, 2A peptides, fluorescent labeling, antennal lobe, optic lobe, neuromuscular junction, *Drosophila*

## Abstract

At synapses, chemical neurotransmission mediates the exchange of information between neurons, leading to complex movement, behaviors, and stimulus processing. The immense number and variety of neurons within the nervous system make discerning individual neuron populations difficult, necessitating the development of advanced neuronal labeling techniques. In *Drosophila*, Bruchpilot-Short and mCD8-GFP, which label presynaptic active zones and neuronal membranes, respectively, have been widely used to study synapse development and organization. This labeling is often achieved via the expression of 2 independent constructs by a single binary expression system, but expression can weaken when multiple transgenes are expressed by a single driver. Recent work has sought to circumvent these drawbacks by developing methods that encode multiple proteins from a single transcript. Self-cleaving peptides, specifically 2A peptides, have emerged as effective sequences for accomplishing this task. We leveraged 2A ribosomal skipping peptides to engineer a construct that produces both Bruchpilot-Short-mStraw and mCD8-GFP from the same mRNA, which we named SynLight. Using SynLight, we visualized the putative synaptic active zones and membranes of multiple classes of olfactory, visual, and motor neurons and observed the correct separation of signal, confirming that both proteins are being generated separately. Furthermore, we demonstrate proof of principle by quantifying synaptic puncta number and neurite volume in olfactory neurons and finding no difference between the synapse densities of neurons expressing SynLight or neurons expressing both transgenes separately. At the neuromuscular junction, we determined that the synaptic puncta number labeled by SynLight was comparable to the endogenous puncta labeled by antibody staining. Overall, SynLight is a versatile tool for examining synapse density in any nervous system region of interest and allows new questions to be answered about synaptic development and organization.

## Teaser

Having access to more efficient tools for visualizing synapses will enable more nuanced research on synaptic development or the causes of synapse dysfunction. SynLight labels both the membranes of neurons as well as their synapses from a single open reading frame with single neuron population specificity, allowing future experimental designs to include additional effectors and markers.

## Introduction

Synapses in the brain facilitate the exchange of information from one neuron to another, culminating in integrated signals that inform the complex computations underlying movement and stimulus sensation. The presynaptic side of the synapse is defined by the active zone, a structural site comprised of quantal release machinery that anchors calcium channels near synaptic vesicles containing neurotransmitter (Wagh *et al.* 2006; Südhof 2012; Ehmann *et al.* 2018). After an influx of calcium through active zone-associated channels following an action potential, neurotransmitter is released into the synaptic cleft where it binds to cognate neurotransmitter receptors on the postsynaptic membrane (Lin and Goodman 1994; Siddiqui and Craig 2011; Wilson 2013; Mosca and Luo 2014; de Ramon Francàs *et al.* 2017). The activation of postsynaptic receptors propagates the signal from the presynaptic neuron to the postsynaptic cell. In the absence of active zones, synaptic communication is drastically impaired or blocked, and function is abrogated. Therefore, the specialized sites of communication between neurons must develop correctly over a specific timeframe and maintain their precise organization throughout an organism's life to ensure that synaptic function continues unabated and retains aspects of synaptic plasticity necessary for appropriate behavioral coordination (Waites *et al.* 2005; Silbereis *et al.* 2016; Farhy-Tselnicker and Allen 2018; Aimino *et al.* 2023). Defects in the development of active zones (both in number and organization) have been found to underlie neurodevelopmental, neuropsychiatric, and even neurodegenerative diseases including autism and schizophrenia, demonstrating the necessity for understanding how synapses develop and organize at the circuit level (Bennett 2011; Grant 2012; Bonansco and Fuenzalida 2016; Mullins *et al.* 2016).

In the central nervous system especially, the high density of synaptic connections and concomitant difficulties of discerning the specific cell or cell type to which an active zone localizes makes the study of synapses with cell-type–specific resolution a challenge. As such, studies using antibodies to active zone machinery are limited in their utility for asking cell-type–specific questions as they recognize synapses in all cells that express active zone proteins in vivo. To better discern how synapses develop and change over time with cell-type specificity, genetic strategies using binary expression systems and transgenic active zone labels have become powerful for studying synaptic organization in identified cell types with high resolution. Work in *Drosophila* especially has contributed greatly to the study of synaptic organization due to the wide variety of genetic tools available for experimental use in synaptic and neuronal labeling (Duhart and Mosca 2022). In recent years, our understanding of synaptic biology has markedly advanced through the study of the active zone protein Bruchpilot, the ortholog of vertebrate ELKS/CAST (Ohtsuka *et al.* 2002), which is an essential presynaptic component at both peripheral and central synapses in the fly (Ohtsuka *et al.* 2002; Wang *et al.* 2002; Wagh *et al.* 2006; Fouquet *et al.* 2009; Südhof 2012). In particular, the transgenic construct, Bruchpilot-Short (Fouquet *et al.* 2009), a truncated version of Bruchpilot that colocalizes with endogenous full-length Bruchpilot, is frequently used to study synaptic organization in specific cell types via binary expression systems like *UAS*/GAL4 (Kremer *et al.* 2010; Berger-Müller *et al.* 2013; Mosca and Luo 2014; Sugie *et al.* 2015; Coates *et al.* 2017, 2020; Mosca *et al.* 2017; Duhart and Mosca 2022; Aimino *et al.* 2023). When Bruchpilot-Short is conjugated to a fluorescent tag and visualized using confocal microscopy, the resultant protein appears as puncta that can be quantified and acts as a proxy measurement for the number of synapses within a specific brain region without interfering with the function or localization of endogenous Bruchpilot or adding ectopic synapses (Wagh *et al.* 2006; Fouquet *et al.* 2009; Kremer *et al.* 2010; Christiansen *et al.* 2011; Mosca and Luo 2014; Coates *et al.* 2017, 2020; Mosca *et al.* 2017; Aimino *et al.* 2023). Use of Bruchpilot-Short has led to novel discoveries about the development and organization of synapses (Fouquet *et al.* 2009; Kremer *et al.* 2010; Christiansen *et al.* 2011; Berger-Müller *et al.* 2013; Mosca and Luo 2014; Coates *et al.* 2017, 2020; Mosca *et al.* 2017; Aimino *et al.* 2023; Parisi *et al.* 2023), demonstrating its effectiveness as a reagent. Bruchpilot-Short is frequently used in conjunction with other synaptic and cellular transgenic constructs to label other synaptic components, including synaptic vesicles (Estes *et al.* 2000; Zhang *et al.* 2002; Williams *et al.* 2019; Certel, McCabe, *et al.* 2022; Certel, Ruchti, *et al.* 2022), other active zone components (Fouquet *et al.* 2009; Liu *et al.* 2011; Mosca *et al.* 2017; Fulterer *et al.* 2018; Özel *et al.* 2019), general cellular architecture (Lee and Luo 1999; Potter *et al.* 2010; Venken, Simpson, *et al.* 2011), and postsynaptic sites (Sánchez-Soriano *et al.* 2005; Leiss, Groh, *et al.* 2009; Leiss, Koper, *et al.* 2009; Kremer *et al.* 2010; Nicolaï *et al.* 2010; Andlauer *et al.* 2014; Mosca and Luo 2014; Mosca *et al.* 2017; Fendl *et al.* 2020; Parisi *et al.* 2023). One such construct, mCD8-GFP (Lee *et al.* 1999), is a membrane-bound GFP tag that, when expressed in any cell type, labels the membranes of that cell, revealing its architecture. When expressed in a particular population of neurons under the control of a binary expression system, mCD8-GFP serves as a general neurite label for both dendrites and axons. Just as Bruchpilot-Short puncta can be quantified as a proxy for the number of synaptic contacts (Fouquet *et al.* 2009; Kremer *et al.* 2010; Christiansen *et al.* 2011; Berger-Müller *et al.* 2013; Mosca and Luo 2014; Coates *et al.* 2017, 2020; Mosca *et al.* 2017; Aimino *et al.* 2023; Parisi *et al.* 2023), membrane markers like mCD8-GFP can be quantified to determine the total volume of neurite membrane in a defined population of neurons (Mosca and Luo 2014; Mosca *et al.* 2017; Aimino *et al.* 2023). Together with Bruchpilot-Short, quantification of puncta number and neurite volume can be expressed as synaptic density within that neuronal population (Mosca and Luo 2014; Mosca *et al.* 2017; Aimino *et al.* 2023). Thus, employing constructs like Bruchpilot-Short-mStraw and mCD8-GFP together makes it possible to ask vital questions about the formation and organization of synapses within a defined population of neurons, thus overcoming previous challenges brought about by the density of synaptic populations in vivo in the central nervous system.

To quantify metrics like synaptic density, it is necessary to express multiple effector or reporter constructs in vivo in tandem as synaptic density is the measure of the number of synaptic puncta within a given volume of neuronal membrane. While increasing the number of simultaneously expressed transgenes enables more complex experimentation, it also carries drawbacks. In cultured cells, multiple plasmids must be cotransfected, potentially resulting in cells that do not incorporate every plasmid that is transfected (González *et al.* 2011). Similarly, driving expression of multiple genes with a binary expression system like GAL4/*UAS* can lead to a dilution of transgene expression, causing effectors and reporters to not function optimally or sufficiently (Brand and Perrimon 1993). Oftentimes, multiple transgenes encoding different effectors and reporters are recombined onto the same chromosome to reduce the number of chromosomes that must be accounted for in the genetic crosses that enable certain experiments. However, this is often time-consuming and challenging, depending on the chromosomal position of each transgene. Further, though it may enable the introduction of additional transgenes and reduce the difficulty of the genetic crosses needed to create the experimental animal, recombination does not reduce the genetic load of the system. Additionally, viral technologies, including AAV-based vectors such as those used in studies of neuronal circuit tracing or as vectors for gene therapy (Naso *et al.* 2017; Weinholtz and Castle 2021), are often limited by the amount of genetic material that can be introduced inside a single viral particle (Grieger and Samulski 2005; Wu *et al.* 2010). As a result, strategies are needed not only to reduce the size of genetic material introduced but also to increase the likelihood of introducing all desired transgenic components with a decreased risk of dilution and failed or weakened expression. One approach to mitigating such drawbacks is to express a single open reading frame that can encode multiple gene products. Initially, internal ribosome entry site (IRES) sequences were used to promote the internal initiation of translation of 2 separate proteins (Martínez-Salas 1999; Douin *et al.* 2004). However, these sequences were limited in their effectiveness as the peptide following the IRES sequence would often have decreased expression compared to the preceding peptide (Kaufman *et al.* 1991; Ye *et al.* 1997; Mizuguchi *et al.* 2000; Underhill *et al.* 2007). More recent work employs 2A viral peptides, which are highly efficient ribosomal skipping peptides (Luke *et al.* 2009; Kim *et al.* 2011; Diao and White 2012; Daniels *et al.* 2014). When incorporated into a particular mRNA, the 2A peptide sequence serves as a skipping site, allowing the ribosome to separate from the mRNA, completing translation of the first sequence, and then re-entering the mRNA at the beginning of the second sequence, starting translation anew and producing a second product. As a result, placing the sequence of a 2A peptide between 2 complete sequences enables 1 continuous mRNA to code for 2 or more polypeptides of interest from a single promoter (Tang *et al.* 2009; Kim *et al.* 2011; Daniels *et al.* 2014). Previous work has indicated that constructs containing 2A peptides are less likely to show the decreased expression of the second protein product, as seen with IRES-containing constructs (Hasegawa *et al.* 2007; Leisegang *et al.* 2008). This approach can decrease the genetic load on a particular system, as now only 1 transgene ensures the expression of multiple products.

In *Drosophila*, there is a multitude of binary expression system drivers (GAL4, lexA, and QF) available, enabling tissue-specific expression in nearly any desired nervous system region and cell type. However, there is considerable variability in the expression strength of binary expression system driver lines, such that not all driver lines can enable transgene expression equivalently. Some cell-type–specific GAL4 lines are weakly expressing, making it difficult to express multiple *UAS* transgenes simultaneously for concurrent labeling of multiple targets. As progressively more *UAS* transgenes are incorporated into an experimental design, the consistent translation of each transgene by a weak GAL4 is less likely, which can result in poor or even failed expression of each respective effector or label. To overcome this expression issue and enable expression of multiple neural markers via a single transgenic construct, we used a vector that contains the 2A peptide from the porcine teschovirus-1 (P2A) coding sequence (Daniels *et al.* 2014) to create a single transgenic construct that encodes both mCD8-GFP and Bruchpilot-Short-mStrawberry (Brp-Short-mStraw) from the same coding sequence. This new construct makes it possible to simultaneously express both neuronal labels from a single ORF rather than 2 independent ORFs (as with 2 independent UAS constructs) while concurrently reducing the genetic load on the system. Therefore, even weakly expressing GAL4s would be able to drive expression of both proteins with high signal fidelity as there would be fewer total transgenes being driven. As the independent constructs fluorescently label synapses as well as the neuronal membrane for visualization at the level of light microscopy, we have named the tool SynLight, a portmanteau of synapse and light. We designed versions of SynLight that can be driven via multiple binary expression systems, including the GAL4/*UAS* and QF/*QUAS* systems, making it possible to ask new questions about the development and organization of synapses throughout the nervous system with less concern over transgenic dilution or failed labeling. Here, we validate SynLight expression in multiple regions of the adult and larval nervous systems in *Drosophila*, including the olfactory, visual, and neuromuscular systems using both the GAL4*/UAS* and QF*/QUAS* systems. We additionally demonstrate that SynLight expression does not affect normal neuronal morphology; active zone puncta number as measurements from SynLight expression are quantitatively indistinguishable from measurements via independent Brp-Short-mStraw or mCD8-GFP transgene expression as well as endogenous Bruchpilot antibody staining. Thus, SynLight labels presynapses and neurite membranes, facilitating their visualization with high resolution and permitting more complex experimental design with reliable quantitative measurements. When expressed in a cell-type–specific manner using existing GAL4 or QF promoter lines, SynLight has a wide applicability to a variety of *Drosophila* nervous system regions and is a versatile tool for studying synaptic development and organization.

## Materials and methods

### Fly stocks and care

All control lines and genetic fly stocks were maintained on cornmeal: dextrose medium (Archon Scientific, Durham, NC, USA) at 21°C while crosses were raised on similar medium at 25°C (unless noted in the text) in incubators (Darwin Chambers, St. Louis, MO, USA) at 60% relative humidity with a 12/12 light/dark cycle.

Transgenes were maintained over balancers with fluorescent markers and visible phenotypic traits to allow for the selection of adults and larvae of the desired genotype. To drive expression in specific classes of CNS neurons, we used the following GAL4 or QF expression lines: *Or47b-GAL4* (Vosshall *et al.* 2000), *Or67d-GAL4* (Stockinger *et al.* 2005), *Or67d-QF* (Liang *et al.* 2013), *Mz19-GAL4* (Jefferis *et al.* 2003), *NP3056-GAL4* (Chou *et al.* 2010), *DIP-γ-GAL4* (Carrillo *et al.* 2015), and *27B03-GAL4* (Jenett *et al.* 2012). Expression at the neuromuscular junction (NMJ) was achieved via *elav^C155^-GAL4* (Lin and Goodman 1994) and *n-syb-QF* (Riabinina *et al.* 2015). The following UAS transgenes were used as synaptic labels or to express molecular constructs for genetic perturbation experiments: *UAS-Brp-Short-mStraw* (Fouquet *et al.* 2009; Mosca and Luo 2014), *UAS-mCD8-GFP* (Lee and Luo 1999), *UAS-SynLight* (*UAS-mCD8-GFP-P2A-Brp-Short-mStraw*; this study). Genotypes for each experiment are listed by figure panel in Table 1.

**Table 1.** Genotypes for each figure panel.

Figure	Panel	Genotype
1	d	*w; Sp/+; UAS-mCD8-GFP-P2A-Brp-Short-mStraw/NP3056-GAL4; +*
	e	*elav^C155^-GAL4/w; CyO/+; UAS-mCD8-GFP-P2A-Brp-Short-mStraw/+; +*
	f	*w; +; QUAS-mCD8-GFP-P2A-Brp-Short-mStraw/n-syb-QF2; +*
2	b–c	*w; Or47b-GAL4/UAS-Brp-Short-mStraw, UAS-mCD8-GFP; +; +*
	d–e	*w; Or47b-GAL4/+; UAS-mCD8-GFP-P2A-Brp-Short-mStraw/+; +*
3	b	*w; +; Or67d-GAL4, UAS-mCD8-GFP-P2A-Brp-Short-mStraw/+; +*
	c	*Or67d-QF/w; +; QUAS-mCD8-GFP-P2A-Brp-Short-mStraw/TM6b, Tb; +*
	d	*w; Mz19-GAL4/+; UAS-mCD8-GFP-P2A-Brp-Short-mStraw/+; +*
	e	*w; Sp/+; UAS-mCD8-GFP-P2A-Brp-Short-mStraw/NP3056-GAL4; +*
4	a, d	*w; +; UAS-mCD8-GFP-P2A-Brp-Short-mStraw/DIP-γ-GAL4; +*
	b	*w; +; UAS-mCD8-GFP-P2A-Brp-Short-mStraw/27B03-GAL4; +*
5	a	*elav^C155^-GAL4/w; Pin or Cyo/+; +; +*
	b, c	*elav^C155^-GAL4/w; Pin or Cyo/+; UAS-mCD8-GFP-P2A-Brp-Short-mStraw/+; +*
	d	*w; +; QUAS-mCD8-GFP-P2A-Brp-Short-mStraw/n-syb-QF2; +*

### Cloning of SynLight plasmid and transgenic lines

Using restriction enzyme cloning, we first inserted the mCD8-GFP sequence (from pC-attB-bursα-mCD8-GFP-T2A-GAL4; a gift from Benjamin White) into a plasmid containing pC5-P2A-KAN (Daniels *et al.* 2014). We used BamHI and Stul as cut sites to put this sequence upstream of the P2A peptide sequence. We subsequently inserted the Bruchpilot-Short-mStrawberry sequence (pENTR-Brp-Short-mStraw; Mosca and Luo 2014), using Nhel and AvrII as cut sites to position the sequence downstream of the P2A peptide and keep all sequences in the same frame of translation. mCD8-GFP-P2A-Bruchpilot-Short-mStrawberry was then migrated from the shuttle vector into a plasmid containing a pUAS-C5-attB sequence using restriction enzyme cloning with Fsel and AscI as the cut sites (to ensure that all sequences remained in the same frame for translation) and ligated together to produce the final plasmid. A similar approach was used to engineer the *QUAS* version of the plasmid (pQUAST-Brp-Short-mStraw-attB; Mosca and Luo 2014). The final plasmid was sequence verified (GeneWiz, South Plainfield NJ), and the final construct sequence is available upon request. A Qiagen Maxi Prep kit (Qiagen, cat. no. 12163) was used to isolate donor plasmid DNA for the creation of transgenic fly lines. Transgenic flies (*UAS* and *QUAS* lines) were generated (BestGene, Chino Hills, CA, USA) with the construct integrated into the attP2 docking site (Groth *et al.* 2004) on the third chromosome. Subsequent transgenic flies (*UAS* line) were also generated (BestGene, Chino Hills, CA, USA) with the construct integrated into the VK00037 docking site (Venken *et al.* 2006) on the second chromosome. After transgenic fly stocks were obtained, construct integration was verified visually by the incorporation of a copy of *mini-white* gene allowing us to identify successful transformants via changes in eye color, as consistent with established transgenic protocols (Brand and Perrimon 1993; Venken, Schulze *et al*. 2011). When the lines were subsequently utilized for experiments, we did not visually observe differences in expression or localization of each marker between the *UAS* transgenes on the second and third chromosomes. As such, for all subsequent experiments, we used the construct on the third chromosome.

### Immunocytochemistry

Adult flies were cleared from vials 1 day before collection and on the following day, newly eclosed adults were chosen based on genotype using identifiable balancers and phenotypic markers. Flies were then aged ten days before dissection and immunostaining. Brains were fixed in 4% paraformaldehyde for 20 min before being washed in phosphate buffer (1× PB) with 0.3% Triton (PBT). Brains were then blocked for an hour in PBT containing 5% normal goat serum (NGS) before being incubated in primary antibodies diluted in PBT with 5% NGS for 2 days at 4°C. Following staining, primary antibodies were discarded and the brains washed 3 × 20 min with PBT and incubated in secondary antibodies diluted in PBT with 5% NGS for an additional 2 days at 4°C. The secondary antibodies were then discarded; the brains were washed 3 × 20 min in PBT and then incubated overnight in SlowFade (Thermo Fisher Scientific, Waltham, MA, USA) gold antifade mounting media and allowed to sink. Brains were then mounted in SlowFade mounting media using a bridge-mount method with no. 1 cover glass shards and stored at 20°C before being imaged (Wu and Luo 2006).

Larvae were processed for immunocytochemistry as previously described (Mosca and Schwarz 2010; Restrepo *et al.* 2022). Larvae were grown in population cages on grape plates with yeast paste until they reached wandering third instar stage. Larval fillet dissections were done in Ca^2+^-free modified *Drosophila* saline, and then fillets were fixed in 4% paraformaldehyde in phosphate-buffered saline (PBS) for 20 min. Samples were then washed with PBS with 0.3% Triton (PBST) for 1 h at room temperature. The fillets were blocked with PBST containing 5% NGS for 1 h at room temperature and then incubated in primary antibodies diluted in PBST with 5% NGS overnight at 4°C. The following day, primary antibodies were discarded, and then fillets were washed with PBST for 3 x 10 min before being placed in secondary antibodies diluted in PBST with 5% NGS for 2 h at room temperature. Fillets were then washed again 5 x 15 min in PBST before being mounted in Vectashield (Vector Laboratories). Samples werestored at 4°C before being imaged.

The following primary antibodies were used: mouse anti-Nc82 (DSHB, cat. no. mAbnc-82, 1:250; Laissue *et al.* 1999), rabbit anti-DsRed (TaKaRa Bio, cat. no. 632496, 1:250; Mosca and Luo 2014), chicken anti-GFP (Aves, cat. no. GFP-1020, 1:1,000; Mosca and Luo 2014), rat anti-N-Cadherin (DSHB, cat. no. mAbDNEX-8, 1:40; Hummel and Zipursky 2004), and Alexa647-conjugated goat anti-HRP (Jackson ImmunoResearch, cat. no. 123-605-021, 1:100; Jan and Jan 1982). Alexa488- (Jackson ImmunoResearch, West Grove, PA, USA), Alexa568- (Thermo Fisher Scientific, Waltham, MA, USA), and Alexa647-conjugated (Jackson ImmunoResearch, West Grove, PA, USA) secondary antibodies were used at 1:250 while FITC-conjugated (Jackson ImmunoResearch, West Grove, PA, USA) secondary antibodies were used at 1:200. In some cases, nonspecific background is recognized by the dsRed antibodies (in the form of large red spots appear around the antennal lobes and outside of the tissue observed). These are part of the background, are not caused by any of the transgenic constructs used (Mosca and Luo 2014), and did not influence any quantification or scoring methods (see below). Additionally, large, bright objects around the antennal lobes can be seen when visualizing projection neurons (PNs) and local interneurons (LNs). These objects correspond to the cell bodies of the neurons being imaged and are likely due to Brp-Short-mStraw beginning to colocalize with endogenous Bruchpilot as it is being synthesized in the ER as well as membrane-bound GFP. This is a known phenomenon associated with Bruchpilot-Short, and as such, these objects are excluded during analysis and do not influence any quantification (see below).

### Imaging and analysis

All images of adult brains were obtained using a Zeiss LSM880 Laser Scanning Confocal Microscope (Carl Zeiss, Oberlochen, Germany) using a 20× 0.8 NA Plan-Apochromat lens, 40× 1.4 NA Plan-Apochromat lens, or a 63× 1.4 NA Plan-Apochromat f/ELYRA lens at an optical zoom of 3×. Images of third instar larval NMJs were obtained using the same confocal microscope using a 40× 1.4 NA Plan-Apochromat lens or a 63× 1.4 NA Plan-Apochromat f/ELYRA lens. Images were centered on the glomerulus or NMJs of interest, and the *z*-boundaries were set based on the appearance of the synaptic labels, Brp-Short-mStraw or mCD8-GFP. Images were analyzed 3 dimensionally using the Imaris Software 9.7.1 (Oxford Instruments, Abingdon, UK) on a custom-built image processing computer (Digital Storm, Fremont, CA, USA) following previously established methods (Aimino *et al.* 2023). For both adult brains and larval NMJs, Brp-Short-mStraw and endogenous Brp puncta were quantified using the “Spots” function with a spot size of 0.6 µm. Neurite volume was quantified using the “Surfaces” function with a local contrast of 3 µm and smoothing of 0.2 µm for *Or47b* olfactory receptor neurons (ORNs). The resultant masks were then visually inspected to ensure their conformation to immunostaining.

### Quantitative measurement and statistical analyses

All data were analyzed using Prism 8 (GraphPad Software, Inc., La Jolla, CA, USA). This software was also used to generate graphical representations of data. Unpaired Student's *t*-tests were used to determine significance between 2 groups while paired Student's *t*-tests were used to determine the significance between puncta numbers for individual NMJs. One-way ANOVA with Tukey's multiple comparisons tests was used to determine significance between groups of 3 or more. A *P*-value of 0.05 was set as the threshold for significance in all studies. For measurements of signal colocalization, Pearson's coefficients and Manders’ split coefficients were obtained using the JACoP: Just Another Co-localization Plugin for ImageJ (Bolte and Cordelières 2006). For each figure, informative genotypes have been presented along with controls appropriate for each genotype.

## Results

### SynLight is designed to label active zones and neurite membranes in the same cell population

**Fig. 1.**
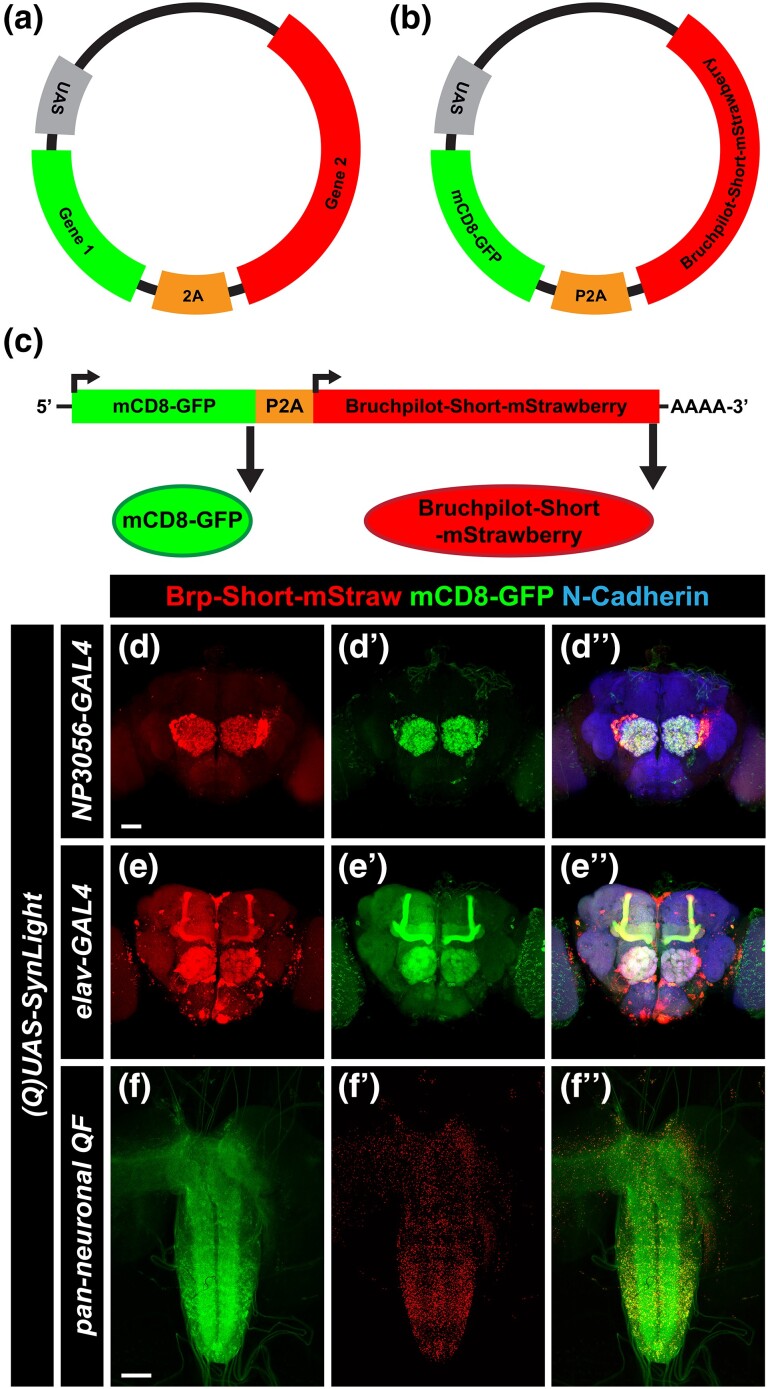
Strategy for generating SynLight, a single transgene that expresses both membrane-tagged GFP and mStrawberry-tagged Bruchpilot-Short. a) Diagram of an example plasmid containing a *UAS* vector and codon-optimized 2A peptide coding sequence (Daniels *et al.* 2014). Flanking either side of 2A is multiple cloning sites and restriction sites that facilitate insertion of 2 or more genes of interest. b) Diagram of the SynLight plasmid. Using restriction enzymes, the mCD8-GFP coding sequence was inserted preceding the P2A coding sequence, and then the Bruchpilot-Short-mStrawberry coding sequence was inserted following the P2A sequence, keeping all sequences in frame. c) Diagram of SynLight mRNA, showing 2 separate proteins being produced from a single mRNA sequence. d–d″) Representative maximum projection confocal image stacks of multiglomerular LNs of the adult brain expressing SynLight and stained with antibodies against mStraw (d), GFP (d″), and N-Cadherin (merge, dʺ). These images show overlapping, yet distinctly different subcellular localization of Brp-Short-mStraw and mCD8-GFP. e–e″) Representative maximum projection confocal image stacks of SynLight being driven pan-neuronally in the adult brain and stained with antibodies as in d. Again, these images show overlapping, yet distinct subcellular localization of Brp-Short-mStraw and mCD8-GFP. f–f″) Representative maximum projection confocal image of the third instar ventral nerve cord expressing SynLight, showing separate endogenous expression of Brp-Short-mStraw (f) and mCD8-GFP (f″) via the native fluorescence from the mStrawberry and GFP fluorophores. Scale bars = 40 μm (d–e); 80 μm (f).

Established approaches in *Drosophila* to examine synapse density in particular classes of neurons typically involve expressing 2 constructs: (1) Brp-Short-mStraw to label active zones made by the neurons and (2) mCD8-GFP (or an equivalent) to label the processes of neurons (Fouquet *et al.* 2009; Kremer *et al.* 2010; Christiansen *et al.* 2011; Berger-Müller *et al.* 2013; Mosca and Luo 2014; Sugie *et al.* 2015; Coates *et al.* 2017, 2020; Mosca *et al.* 2017; Aimino *et al.* 2023; Parisi *et al.* 2023). Previous work established that 2A peptide approaches work efficiently in *Drosophila* for encoding multiple proteins from a single ORF (Diao and White 2012; Daniels *et al.* 2014). Therefore, we designed and built a single construct containing Brp-Short-mStraw and mCD8-GFP separated by a 2A peptide and named it SynLight. SynLight takes advantage of P2A, a viral 2A peptide from porcine teschovirus-1 (Kim *et al.* 2011; Daniels *et al.* 2014). The P2A protein is derived from a ribosomal skipping protein that allows multiple separate proteins to be made from a single mRNA (Tang *et al.* 2009; Kim *et al.* 2011). Using a vector that contains the P2A coding sequence, multiple cloning sites, and restriction sites (Le *et al.* 2007; Daniels *et al.* 2014), we engineered a single transgene that produces multiple proteins from a single coding sequence via restriction cloning (Fig. 1a). The resultant vector contained mCD8-GFP inserted into the first restriction site and Brp-Short-mStraw inserted into the second site with the 2 separated by P2A, producing *mCD8-GFP-P2A-Brp-Short-mStraw* (Fig. 1b). From the single mRNA produced by the transgene following activation by a promoter driver line, 2 separate proteins would be produced, Brp-Short-mStraw and mCD8-GFP (Fig. 1c). We engineered both *UAS* and *QUAS* versions of SynLight and established transgenic lines on the third chromosome in the attP2 site (Groth *et al.* 2004) so that the construct could be used with multiple binary expression systems. We subsequently established a transgenic line on the second chromosome in the VK00037 site (Venken *et al.* 2006). When SynLight was expressed in olfactory neurons of the adult brain using *NP3056-GAL4* (Fig. 1d–dʺ; Chou *et al.* 2010) or pan-neuronally using *elav^C155^-GAL4* (Fig. 1e–eʺ; Lin and Goodman 1994) after immunohistochemical staining for Brp-Short-mStraw and mCD8-GFP as established previously (Mosca and Luo 2014; Mosca *et al.* 2017; Aimino *et al.* 2023), we observed clear, subcellularly distinct signal for both Brp-Short-mStraw and mCD8-GFP. We obtained similar findings when SynLight was expressed in the ventral nerve cord of third instar larvae using the pan-neuronal QF driver *n-syb-QF* and visualized using the native fluorescence of both labels (Fig. 1f–fʺ; Riabinina *et al.* 2015). For both the central and peripheral nervous systems, these data demonstrated that there was separation between the 2 products and indicated that ribosomal skipping occurred successfully during translation, resulting in the synthesis of Brp-Short-mStraw and mCD8-GFP separately (insufficient separation would manifest as precise overlap between the mCD8-GFP and Brp-Short-mStraw channels). With the successful establishment of transgenic *UAS-* and *QUAS-SynLight* lines, we further sought to validate the construct as a synaptic labeling and quantification method.

### Quantification of synapses and neuronal morphology in antennal lobe neurons using SynLight

Both Brp-Short-mStraw and mCD8-GFP have enabled quantitative analyses of synaptic organization at peripheral and central synapses, resulting in established measurements of synapse number and neurite volume, especially in the olfactory system (Kremer *et al.* 2010; Christiansen *et al.* 2011; Mosca and Luo 2014; Mosca *et al.* 2017; Aimino *et al.* 2023). As such, there is a rich history of control data against which we can benchmark SynLight performance. To demonstrate the utility of SynLight for making quantitative measurements of synapse organization and density, we first turned to ORNs in the *Drosophila* antennal lobe. In the antennal lobe, ORNs, PNs, and LNs are the 3 major neuron types that contribute to the sensation and subsequent relay of olfactory information to higher-order brain structures such as the mushroom bodies and the lateral horn (Vosshall *et al.* 2000; Jefferis *et al.* 2001; Tanaka *et al.* 2009). ORNs, PNs, and LNs each project to the roughly 50 glomeruli that comprise the antennal lobe, which are subdivided based on the type of olfactory information they receive, and form synapses with each other to create functional circuits (Suh *et al.* 2004; Hallem and Carlson 2006; Jefferis *et al.* 2007; Grabe and Sachse 2018).

**Fig. 2.**
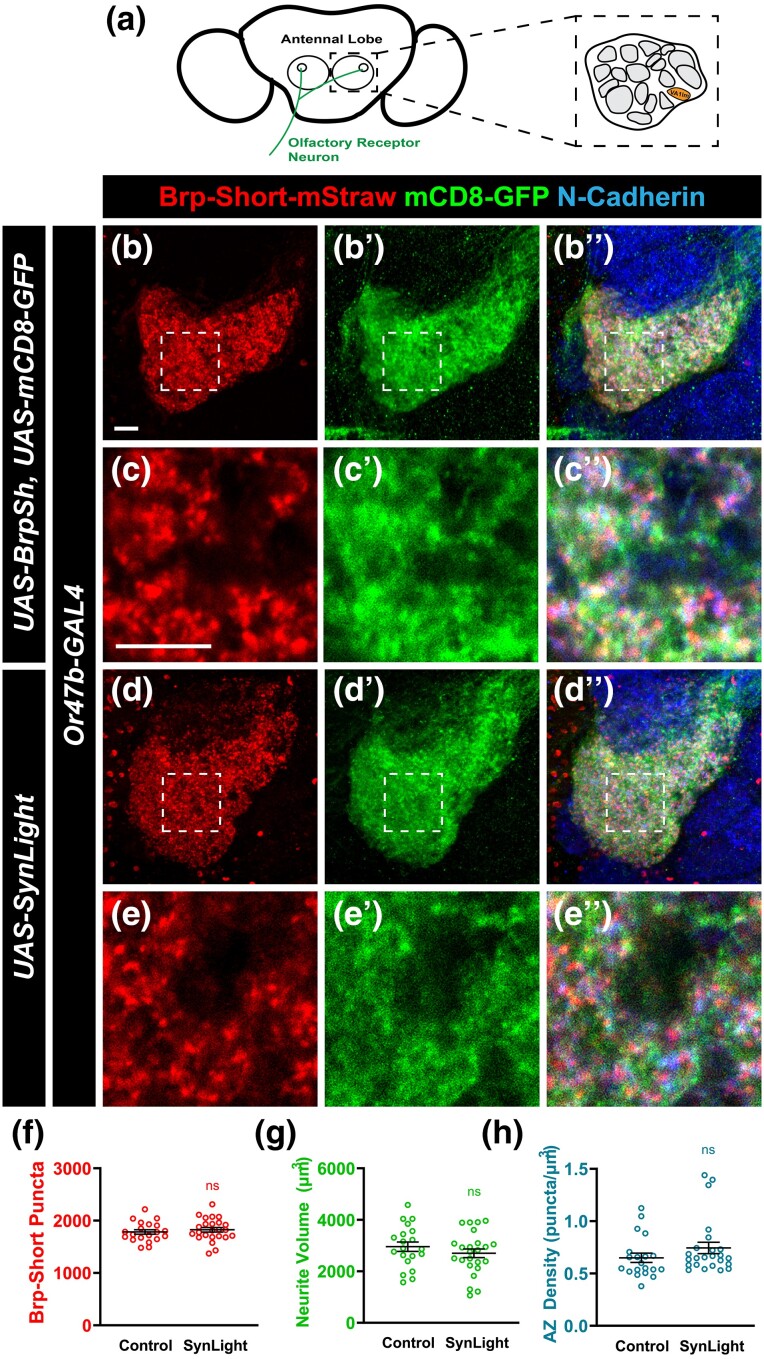
SynLight expression does not affect synapse number in olfactory neurons. a) Diagram of the *Drosophila* antennal lobes showing ORNs (green) of the VA1lm glomerulus (orange). b–b″) Representative confocal image stacks of 10-day-old male adult VA1lm ORNs expressing Brp-Short-mStraw and membrane-tagged GFP separately and stained with antibodies against mStraw (b), GFP (b″), and N-Cadherin (merge, bʺ). c–c″) High-magnification, single optical image section of ORNs from inset in b showing colocalization, but not complete overlap, of synaptic labels. d–d″) Representative confocal image stacks of 10-day-old male adult VA1lm ORNs expressing SynLight and stained with antibodies as in b. e–e″) High-magnification, single optical image section from inset in d also showing colocalization, but not complete overlap, consistent with subcellular localization and suggesting P2A-mediated cleavage is occurring successfully. f–h) Quantification of Brp-Short-mStraw puncta number f), membrane GFP volume g), and synapse density h) for adult male VA1lm ORNs expressing either SynLight or Brp-Short-mStraw and membrane-tagged GFP separately. Brp-Short-mStraw puncta number, neurite volume, and synapse density obtained using SynLight are not significantly different from using Brp-Short-mStraw and membrane-GFP separately. For each genotype, *n* ≥ 20 glomeruli from 10 brains. n.s., not significant. Scale bars = 5 μm.

We first examined ORNs of the VA1lm glomerulus (Fig. 2a) using *Or47b-GAL4* (Vosshall *et al.* 2000) and compared expression of independent Brp-Short-mStraw and membrane-bound GFP transgenes (Fig. 2b–cʺ) to SynLight (Fig. 2d–eʺ) following immunohistochemical staining for Brp-Short-mStraw and mCD8-GFP. Qualitatively, expression patterns and subcellular localization of SynLight vs mCD8-GFP/Brp-Short-mStraw from independent transgenes were indistinguishable from one another regardless of genotype. Subsequently, for each genotype, we quantified Brp-Short-mStraw puncta and neurite volume (as represented by mCD8-GFP staining) and found that Brp-Short-mStraw puncta number (Fig. 2f) and neurite volume (Fig. 2g) in VA1lm ORNs were not significantly different between flies expressing SynLight and those expressing Brp-Short-mStrawberry and mCD8-GFP independently. We then calculated synapse density (Fig. 2h) by dividing the Brp-Short-mStraw puncta number by the neurite volume for each individual glomerulus and continued to find no significant difference between ORNs expressing SynLight and those expressing Brp-Short-mStraw and mCD8-GFP independently. This indicates that SynLight accurately recapitulates independent Brp-Short and mCD8-GFP expression both qualitatively and quantitatively. Moreover, SynLight expression does not interfere with synaptic organization or development of individual neuron types, as the mature synapse number and volume are unaltered when compared to published data (Mosca and Luo 2014; Mosca *et al.* 2017; Aimino *et al.* 2023). Therefore, SynLight is a viable strategy for quantitatively assessing synaptic organization with fewer genetic transgenes.

**Fig. 3.**
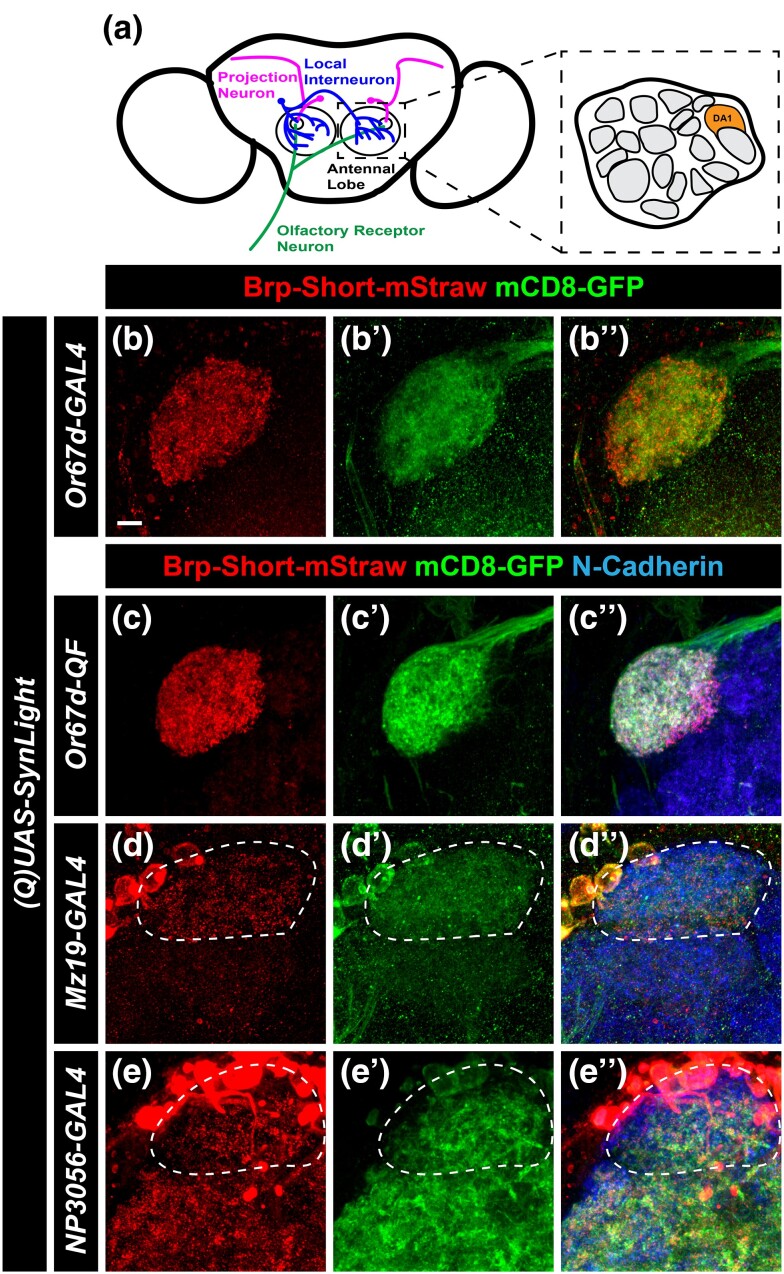
SynLight labels presynaptic active zones and neuronal membranes in multiple cell types of the olfactory system. a) Diagram of the *Drosophila* antennal lobes showing ORNs (green), PNs (magenta), and multiglomerular LNs (blue) of the DA1 glomerulus (orange). b–c″) Representative confocal image maximum projections of male adult DA1 ORNs expressing SynLight via a GAL4 b) or QF c) driver and stained with antibodies against mStraw (b, c), GFP (b″, c″), and N-Cadherin (merge, bʺ, cʺ). d–e″) Representative confocal image maximum projections of male adult DA1 PNs (dashed white lines) d) and multiglomerular LNs e) of the DA1 glomerulus (dashed white lines) expressing SynLight and stained with antibodies as in b–c. In e, multiglomerular LNs project throughout the antennal lobe but only the DA1 glomerulus is encircled for comparison. For each experimental group, *n* ≥ 5 brains. Scale bar = 5 μm.

Having established that SynLight is robustly expressed in antennal lobe VA1lm ORNs without affecting synaptic organization, we next expanded our analysis by driving SynLight expression in multiple cell types of the olfactory system (Fig. 3a). When driven in a different population of antennal lobe ORNs using *Or67d-GAL4* (DA1 ORNs; Stockinger *et al.* 2005) or *Or67d-QF* (Liang *et al.* 2013), we saw robust labeling of ORN active zones and neurites (Fig. 3b–cʺ). We also examined SynLight expression in other antennal lobe neurons beyond ORNs: we used *Mz19-GAL4* (Jefferis *et al.* 2003) and *NP3056-GAL4* (Chou *et al.* 2010) to drive SynLight expression in DA1 PNs (Fig. 3d–dʺ) and multiglomerular LNs of DA1 (Fig. 3e–eʺ), respectively. As with DA1 ORNs, we found that SynLight labels active zones and neurites in both classes of neurons and that the labeling is consistent with previous results from the same drivers (Mosca and Luo 2014; Aimino *et al.* 2023). Taken together, SynLight expression is evident regardless of the olfactory neuron class in which it is expressed or via which binary expression system it is driven, further demonstrating its utility as a tool for studying synapse formation and organization.

### SynLight labels presynaptic connections in neurons of the visual system

**Fig. 4.**
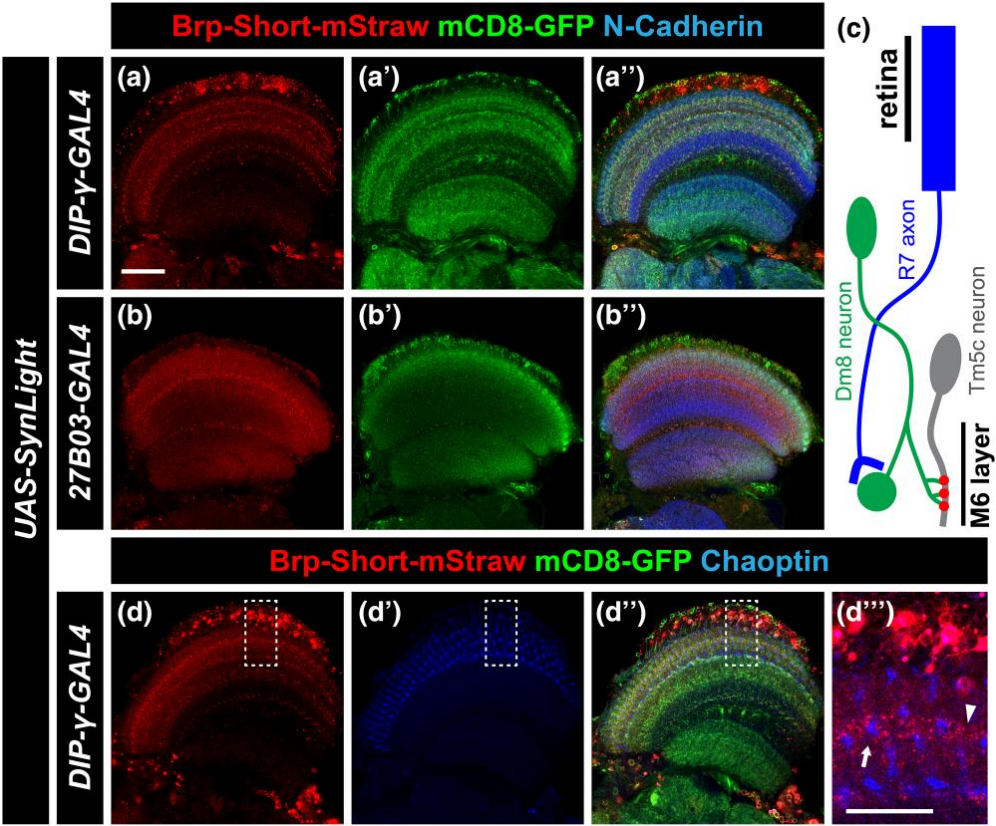
SynLight labels presynaptic active zones and neuronal membranes in neurons of the visual system. a–b″) Representative single confocal image sections of male adult brains expressing SynLight using *DIP-γ-GAL4* to label Dm8 neurons a) or *27B03-GAL4* to label optic lobe neurons b) and stained with antibodies against mStraw (a, b), GFP (a″, b″), and N-Cadherin (merge, aʺ, bʺ). c) Schematic showing the connections between R7 photoreceptor axons (labeled), Dm8 neurons (labeled), and Tm5c neurons (labeled). R7 axons project from the retina and synapse onto the dendrites of Dm8 neurons. Dm8 neurons subsequently form synapses with Tm5c neurons, forwarding the visual information received from R7 axons. The presynaptic active zones of Dm8 neurons (d) and axon terminals of R7 cells (d″) are both found in the M6 layer of the medulla. d–d″) Representative single confocal image sections of male adult brains expressing SynLight in Dm8 neurons and stained with antibodies against mStraw (d), GFP (merge, dʺ), and Chaoptin (d″). dʺ′) Single, high-magnification image section from insets (dashed boxes, d) showing mStraw and Chaoptin costaining. Arrow indicates region of Brp-Short-mStraw and Chaoptin in close proximity while arrowhead indicates a region with only Brp-Short-mStraw. For each experimental group, *n* ≥ 5 brains. Scale bars = 20 μm a); 10 μm (dʺ′).

To expand our study of SynLight expression beyond the olfactory system, we next examined the fly visual system. Both the anatomy and organization of the fly visual system have been well characterized (Takemura *et al.* 2013, 2015; Yang and Clandinin 2018; Scheffer *et al.* 2020; Choi *et al.* 2021), and the fly visual system represents an excellent model for studying synaptic development and organization (Clandinin and Zipursky 2002) as well as visual processing (Yang and Clandinin 2018). Further, tagged versions of Bruchpilot have been used extensively to characterize both the cellular events underlying and the molecular mechanisms supporting, synaptic plasticity in the visual system (Berger-Müller *et al.* 2013; Chen *et al.* 2014; Sugie *et al.* 2015; Shimozono *et al.* 2019; Araki *et al.* 2020; Kawamura *et al.* 2020; Duhart and Mosca 2022; Osaka *et al.* 2023), highlighting the utility of Brp-based labeling tools in understanding visual biology. To determine if SynLight could be used to concurrently label neuronal membranes and active zones in the visual system, we drove *UAS-SynLight* in the visual system using 2 different GAL4 drivers, *Dpr Interacting Protein-γ (DIP-γ)-GAL4* (Carrillo *et al.* 2015) and *27B03-*GAL4 (Jenett *et al.* 2012) and examined both Brp-Short puncta and GFP-tagged neuronal membranes. *DIP-γ-GAL4* labels Dm8 neurons in layer M6 of the medulla (Fig. 4a–aʺ) while *27B03-GAL4* drives expression in neurons of the optic lobe (Fig. 4b–bʺ). In both cases, we observed robust expression of SynLight and labeling consistent with release sites (via Brp-Short-mStraw) and general neuronal processes (via mCD8-GFP), indicating the efficacy and applicability of the SynLight construct beyond the olfactory system.

The Dm8 neurons labeled by *DIP-γ-GAL4* are postsynaptic to R7 photoreceptor neurons and form a connection analogous to the ORN to PN synapses in the antennal lobe (Fig. 4c; Takemura *et al.* 2013). Processes of Dm8 neurons also form synaptic contacts onto Tm5c neurons, comprising a circuit that mediates UV preference (Karuppudurai *et al.* 2014). Both connections (R7 → Dm8 and Dm8 → TM5c) form within the M6 layer of the medulla, suggesting that presynaptic R7 terminals should localize near (but not overlap with) Dm8 presynaptic terminals. We reasoned that concurrent labeling of R7 and Dm8 terminals would result in presynaptic staining of both neuron classes and that their respective presynaptic sites would be found in close proximity to one another within layer M6 of the medulla. To do so, we drove expression of SynLight in Dm8 neurons and costained the optic lobes with antibodies to Chaoptin, a marker for R7 photoreceptor cells (Krantz and Zipursky 1990). Indeed, when we specifically examined the M6 layer, we found that Dm8 Brp-Short puncta and R7 photoreceptor Chaoptin are present in similar regions of the optic lobe (Fig. 4d–dʺ). Furthermore, Dm8 Brp-Short-mStraw puncta and R7 Chaoptin signals do not overlap but are instead adjacent to one another as predicted (Fig. 4dʺ′). Thus, SynLight expression can recapitulate expected patterns of synaptic organization in the visual system, indicating its utility as a synaptic label. Taken together with our findings from the olfactory system (Figs. 2 and 3), these data show that SynLight is a robust, reliable tool for concurrent labeling of synaptic active zones and general neuronal processes in multiple central nervous system populations.

### SynLight accurately labels and quantifies active zones at neuromuscular synapses

**Fig. 5.**
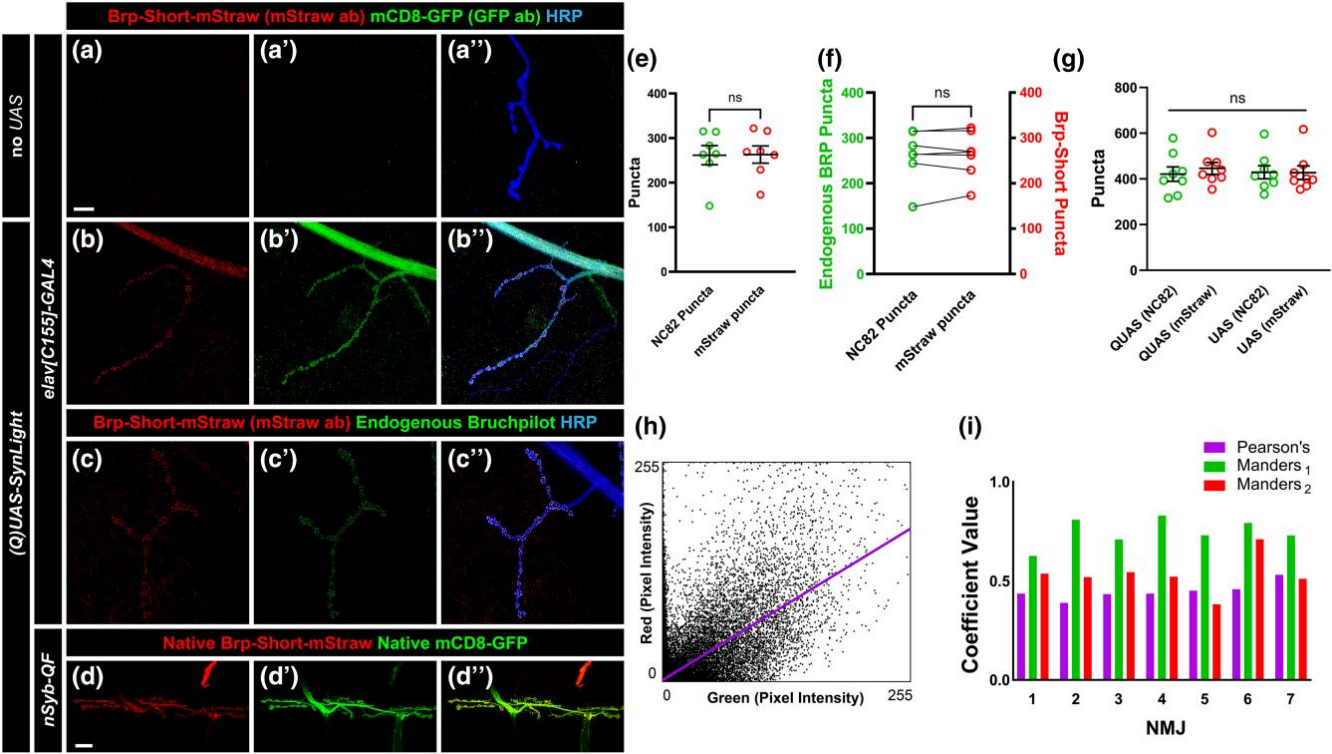
SynLight labels the larval NMJ and does not alter synapse formation. a–b″) Representative confocal image maximum projections of muscle 4 NMJs in control a) or SynLight-expressing b) wandering third instar larvae stained with antibodies against mStraw (a, b), GFP (a″, b″), and HRP (merge, aʺ, bʺ). The negative control lacking SynLight shows no mStraw or GFP immunoreactivity while pan-neuronal SynLight expression shows clear visibility of both markers. c–c″) Representative confocal image maximum projections of a muscle 4 NMJ expressing pan-neuronal SynLight and stained for antibodies against mStraw (c), NC82 (c″), and HRP (merge, cʺ). d–d″) Representative confocal image maximum projections of muscle 6/7 NMJs expressing SynLight showing endogenous expression of Brp-Short-mStraw (d) and mCD8-GFP (d″) via native fluorescence from the mStrawberry and GFP fluorophores. e) Quantification of active zone puncta visualized by antibody staining of endogenous Bruchpilot (via monoclonal antibody NC82) or expression of Brp-Short-mStraw via SynLight from c. There is no significant difference between Brp-Short-mStraw-positive and NC82-positive puncta. f) Quantification of active zone puncta as in e with paired comparisons for each individual NMJ. These data corroborate that, for each individual NMJ, there is no significant difference between Brp-Short-mStraw-positive and NC82-positive puncta number. g) Quantification of active zone puncta number visualized by antibody staining of endogenous Bruchpilot or expression of Brp-Short-mStraw via SynLight as in e for muscle 4 NMJ terminals expressing either *QUAS-SynLight* [QUAS (NC82) and QUAS (mStraw)] or *UAS-SynLight* [UAS (NC82) and UAS (mStraw)]. There is no significant difference between NC82 and Brp-Short-mStraw puncta number when either SynLight variant (*QUAS* or *UAS*) is used. h) Representative scatterplot of green pixel intensity (from NC82 puncta signal) and red pixel intensity (from Brp-Short-mStraw puncta signal) from a single muscle 4 NMJ terminal. Pearson's coefficient (first column in purple) shows a positive correlation between the 2 signals, suggesting colocalization. i) Correlation of active zone puncta visualized by antibody staining of endogenous Bruchpilot (via monoclonal antibody NC82) to active zone puncta expression of Brp-Short-mStraw via SynLight from each individual terminal in c. Correlations are represented by Pearson's coefficients (purple) or Manders’ split coefficients (M_1_, second column in green; M_2_, third column in red). Positive Pearson's coefficient indicates positive correlation between NC82 puncta and Brp-Short-mStraw puncta. Values approaching 1 for Manders’ coefficients indicate a similar correlation between the Brp-Short and NC82 signals. For each experimental group, *n* ≥ 7 NMJs. n.s., not significant. Scale bars = 15 μm a); 20 μm d).

To explore the utility of SynLight beyond the central nervous system, we next turned to peripheral NMJ synapses. NMJ synapses are highly stereotyped and are a long-studied, powerful system for uncovering active zone biology (Landgraf and Thor 2006; Menon *et al.* 2013; Chou *et al.* 2020) making them an optimal synapse for examining SynLight expression and quantification. We first expressed SynLight pan-neuronally via *elav^C155^-GAL4* (Lin and Goodman 1994) and observed robust labeling of both general membranes (via mCD8-GFP) and active zones (via Brp-Short-mStraw) at NMJs (Fig. 5b–b″) that was absent from nonexpressing controls (Fig. 5a–a″). Consistently, mStraw-positive Brp-Short puncta labeled by SynLight overlapped with endogenous Bruchpilot antibody staining (Fig. 5c–c″), suggesting that SynLight labeling accurately revealed endogenous active zones. We further observed similar Brp-Short-mStraw and mCD8-GFP expression and localization with *QUAS-SynLight* (Fig. 5d–d″) expression via *n-syb-QF* (Riabinina *et al.* 2015), indicating that multiple binary expression system versions of SynLight provide robust labels. Taken together, this indicates that SynLight is effectively and accurately expressed at NMJ synapses in separable pools reflecting membranes and release sites.

Having established that SynLight accurately localizes to NMJ synapses and membranes, we next assessed SynLight as a quantitative tool for active zone puncta. NMJ terminals have a characteristic number of active zone puncta when stained with antibodies to endogenous Bruchpilot, highlighting this metric as a reliable quantitative measurement of synaptic growth (Collins and DiAntonio 2007; Daniels *et al.* 2008; Wairkar *et al.* 2008). To determine if SynLight could be reliably used to quantify active zones, we counted Brp-Short-mStraw puncta at muscle 4 NMJ terminals and compared the data to counts of puncta recognized by the monoclonal antibody NC82 to endogenous Bruchpilot (Laissue *et al.* 1999; Wagh *et al.* 2006). There was no significant difference in the average puncta number visualized by mStraw (via SynLight) or NC82 (monoclonal antibody to Brp) staining (Fig. 5e), suggesting that SynLight could accurately quantify Brp-positive endogenous active zone number. To further validate our approach, we compared the individual Brp-Short-mStraw and NC82 puncta counts for each muscle 4 NMJ terminal to ensure that each metric gave the same result at the single NMJ being examined. Further, we observed no significant difference between the paired Brp-Short-mStraw and NC82 puncta number for each individual NMJ (Fig. 5f), demonstrating accurate and congruent reporting. To ensure that variants of *SynLight* for different binary expression systems (namely, GAL4 vs QF) function equivalently, we also compared Brp-Short-mStraw puncta number and NC82 puncta number between muscle 4 NMJ terminals expressing either *UAS-SynLight* or *QUAS-SynLight*. We found that there was no significant difference between the average Brp-Short-mStraw puncta number and the average NC82 puncta number (Fig. 5g), regardless of which SynLight variant was used to visualize these NMJs.

Although we observed no difference in average Brp-Short-mStraw or NC82 puncta number, we sought to further assess the utility of SynLight in accurately labeling presynaptic active zones using colocalization analysis. We analyzed signal colocalization between the Brp-Short-mStraw and NC82 channels for each individual muscle 4 NMJ terminal image used in Fig. 5c, e, and f. We plotted pixel intensity for each fluorescence signal and observed a positive correlation between NC82 signal and Brp-Short-mStraw signal (Fig. 5h), suggesting colocalization between the 2 labels. We also calculated both Pearson's and Manders’ split coefficients for each muscle 4 NMJ terminal to more directly assess colocalization (Fig. 5i). Pearson's coefficients range from 1 to −1 with a value of 1 indicating full colocalization and a value of −1 indicating no correlation between the 2 signals (Manders *et al.* 1993). Manders’ coefficients range from 0 to 1 with a value of 1 indicating complete cooccurrence of pixel signal (Manders *et al.* 1993). For each muscle 4 NMJ terminal, we obtained positive Pearson's coefficients and Manders’ coefficients close to 1, implying correlation of Brp-Short-mStraw and NC82 puncta signal and colocalization of our synaptic labels (Fig. 5i; average *P* = 0.447, average *M*_1_ = 0.746, and average *M*_2_ = 0.53). In all, the data indicate that SynLight accurately reports NMJ synaptic organization both qualitatively and quantitatively. Combined, our findings establish that SynLight functions as a robust synaptic label at both peripheral and central synapses in *Drosophila*.

## Discussion

As technologies improve, making novel manipulations of, and labeling in, the nervous system possible, there is a growing need to incorporate more genetic components into experiments. Experiments in model organisms especially often have at least 3 transgenes (Venken, Schulze *et al*. 2011; Chou *et al.* 2020; Duhart and Mosca 2022) for even basic experiments: a genetic driver (e.g. GAL4, QF, lexA, and Cre), a reporter (GFP, synaptic labels, and receptor labels), and an effector (e.g. an optogenetic regulator of activity, toxin, endocytosis blocker, and kinase activity regulator). Multiple challenges exist, however, with such experiments. First, each transgene must be accounted for in genetic crosses to obtain experimental animals, leading to complex crosses where it is increasingly challenging to obtain “correct” progeny based on Mendelian ratios and unanticipated lethality. Second, genetic driver strength can be diluted by multiple transgenes (Brand and Perrimon 1993), leading to increased variability of expression and/or reduced efficacy of expressed transgenes. Finally, space constraints (from chromosome number or limits on viral DNA payload) can limit the number of genetic transgenes that can be present in the final experimental animal. Though some approaches like recombination of multiple transgenes onto the same chromosome can increase available genetic space for other transgenes and alleviate some of these concerns, recombinants do not reduce the total number of transgenes and the “genetic load” of the system persists. To begin to address some of these concerns, we developed a new strategy, SynLight, that uses the viral P2A ribosomal skipping peptide (Diao and White 2012; Daniels *et al.* 2014) to produce a single transgene that expresses both the membrane label mCD8-GFP (Lee *et al.* 1999) and the active zone label Brp-Short-mStraw (Fouquet *et al.* 2009; Mosca and Luo 2014). We demonstrate that this strategy is effective in multiple central and peripheral neurons and is quantitatively similar to synaptic measurements using independent mCD8-GFP or Brp-Short-mStraw expression alone (Mosca and Luo 2014; Aimino *et al.* 2023). Using SynLight in either CNS or PNS experiments will enable more complex studies in vivo without sacrificing the number of labels possible.

To develop a construct for use in *Drosophila* that encodes both a presynaptic active zone marker as well as a neuronal membrane tag from a single sequence, we incorporated the P2A peptide, a ribosomal skipping sequence (Luke *et al.* 2009). This virus-derived peptide sequence mediates a skipping event during translation that enables the production of both proteins from a single mRNA (Tang *et al.* 2009; Kim *et al.* 2011; Daniels *et al.* 2014). We incorporated the established fly active zone label Brp-Short-mStraw (Kittel *et al.* 2006; Wagh *et al.* 2006; Fouquet *et al.* 2009; Kremer *et al.* 2010; Christiansen *et al.* 2011; Mosca and Luo 2014; Duhart and Mosca 2022; Aimino *et al.* 2023) and the general membrane marker mCD8-GFP (Lee *et al.* 1999) to produce a single SynLight transgene that concurrently labels all neuronal membranes via mCD8-GFP and mature active zones via Brp-Short. We established multiple SynLight transgenic constructs (Fig. 1) on the second and third chromosomes for *GAL4/UAS* expression (Brand and Perrimon 1993) and on the third chromosome for *QF/QUAS* expression (Potter *et al.* 2010). We further demonstrated that SynLight is expressed with high fidelity in multiple classes of *Drosophila* CNS neurons, including those of the olfactory (Figs. 2 and 3) and visual (Fig. 4) systems. Further, the 2 products, mCD8-GFP and Brp-Short-mStraw, are readily separable in all neurons and, when quantified, produce similar results to established data and to expression of individual analogous constructs alone (Fig. 2). Thus, SynLight is effective for quantifying synapse density with only 1 construct, whereas previous experiments required 2 independent transgenes. We also demonstrated similar utility for SynLight at peripheral NMJ synapses. Not only is expression robust and labeling evident (Fig. 5) for membranes and active zones; measurements with SynLight accurately recapitulate data obtained from established antibodies to endogenous Bruchpilot (Laissue *et al.* 1999; Wagh *et al.* 2006). In all, SynLight accurately labels multiple subcellular structures via only 1 transgene.

Tools like SynLight will allow greatly increased utility within the fly nervous system. This will not only promote more complex and nuanced questions but also reduce experimental work. For example, to determine whether reduction of the function of a single gene influences synaptic density, 5 transgenes would optimally be required: a genetic GAL4/QF/lexA driver, an RNAi transgene to reduce specific gene function, Dcr2 to increase RNAi efficacy (Dietzl *et al.* 2007), mCD8-GFP (or an equivalent membrane marker) to measure neurite volume, and Brp-Short (or an equivalent active zone label) to quantify release sites. Not only is this a genetically complex experiment, but it may also reduce expression when a driver is used to express 4 independent transgenes. While the expression level tendered by strong drivers will enable the experiment, many circumstances will result in either reduced expression of the labels and/or reduced efficacy of the RNAi, leading to difficulty in interpreting the results. Previous approaches (Mosca *et al.* 2017; Aimino *et al.* 2023) have expressed the mCD8-GFP and Bruchpilot-Short transgenes in separate experiments, but as the measurements are then not taken from the same animal, synaptic density is not directly calculable. SynLight circumvents that issue by using only a single transgene to express both labels, thus increasing the utility of available experiments. Beyond simple perturbation experiments using a single class of neurons, SynLight also enables more complex, transsynaptic questions. When multiple binary expression systems are needed to label and manipulate different neuronal populations, as with pre- and postsynaptic neurons (Mosca and Luo 2014; Parisi *et al.* 2023), the required experiments must be carefully designed with limits on the ensuing number of transgenes (since multiple genetic drivers now contribute to the genetic load). In this case, 1 expression system would drive the expression of synaptic labels in 1 neuronal population while another expression system would drive an effector transgene in a second neuronal population. Employing a construct such as SynLight, which codes for multiple proteins from a single sequence, reduces the genetic load on the system and makes it easier to produce the correct experimental fly stocks in the absence of genetic dilution. Additionally, this transgene can be recombined with other transgenes, further simplifying the creation of a desired stock. Finally, our use of SynLight presents further proof-of-principle of the utility of 2A peptides in vivo in *Drosophila*. Prior work established transgenes containing a Ca^2+^ reporter like GCaMP and a membrane label (Daniels *et al.* 2014). The use of T2A to produce GAL4-expressing constructs at the end of a protein reporter or endogenous protein has also greatly enhanced neuronal circuit study (Diao and White 2012; Lee *et al.* 2018; Kondo *et al.* 2020). By including 2 different reporters for membranes and active zones, this greatly increases the number and kinds of experiments possible. Future versions can pair effectors and labels (like Brp-Short-mStraw and an activity-altering construct) or even enzymes and labels (like a FLPase and Brp-Short-mStraw) as needed to design different kinds of experiments. Overall, SynLight enables the high-resolution visualization of presynaptic active zones as well as neuronal membranes in vivo via a single transgene, thus reducing the genetic load on the system. We anticipate that this new approach will be applied throughout the central and peripheral nervous system to answer more complicated questions about circuit biology, neurodevelopment, and synaptic organization.

## Supplementary Material

jkad221_Supplementary_Data

## Data Availability

All fly lines have been deposited with the Bloomington *Drosophila* Stock Center and are also available upon request. The final plasmids for all variants of SynLight have been deposited with the *Drosophila* Genetics Resource Center. The final construct sequence for SynLight is included in the online Supplementary material. Supplemental material available at G3 online.
