# Supplementary material for: SynLight: a bicistronic strategy for simultaneous active zone and cell labeling in the Drosophila nervous system
Source: G3 (Bethesda). 2023 Sep 27;13(11):jkad221. doi: 10.1093/g3journal/jkad221 (PMC10627267; doi:10.1093/g3journal/jkad221)
Supplement: jkad221_Supplementary_Data [file jkad221_supplementary_data.docx]

**S1.** Construct sequence for UAS-mCD8-GFP-P2A-Brp-Short-mStraw

cggagtactgtcctccgagcggagtactgtcctccgagcggagtactgtcctccgagcggagtactgtcctccgagcggagtactgtcctccgagcggagactctagcgagcgccggagtataaatagaggcgcttcgtctacggagcgacaattcaattcaaacaagcaaagtgaacacgtcgctaagcgaaagctaagcaaataaacaagcgcagctgaacaagctaaacaatctgcagtaaagtgcaagttaaagtgaatcaattaaaagtaaccagcaaccaagtaaatcaactgcaactactgaaatctgccaagaagtaattattgaatacaagaagagaactctgaatagggaattgggaattggccggcctacttaattaagtggaattcaaaggtacccgcccgggatcagatccgcggccgcataggccactagtggatctggatccatggccagccccctgacccgcttcctgagcctgaacctgctgctgctgggcgagagcatcatcctgggcagcggcgaggccaagccccaggcccccgagctgcgcatcttccccaagaagatggatgccgagctgggccagaaggtggatctggtgtgcgaggtgctgggcagcgtgagccagggctgcagctggctgttccagaacagcagcagcaagctgccccagcccaccttcgtggtgtacatggccagcagccacaacaagatcacctgggatgagaagctgaacagcagcaagctgttcagcgccatgcgcgataccaacaacaagtacgtgctgaccctgaacaagttcagcaaggagaacgagggctactacttctgcagcgtgatcagcaacagcgtgatgtacttcagcagcgtggtgcccgtgctgcagaaggtgaacagcaccaccaccaagcccgtgctgcgcacccccagccccgtgcaccccaccggcaccagccagccccagcgccccgaggattgccgcccccgcggcagcgtgaagggcaccggcctggatttcgcctgcgatatctacatctgggcacccctggccggcatctgcgtggccctgctgctgagcctgatcatcaccctgatctgctaccacagccgcggcagcatggtgagcaagggcgaggagctgttcaccggggtggtgcccatcctggtcgagctggacggcgacgtaaacggccacaagttcagcgtgtccggcgagggcgagggcgatgccacctacggcaagctgaccctgaagttcatctgcaccaccggcaagctgcccgtgccctggcccaccctcgtgaccaccctgacctacggcgtgcagtgcttcagccgctaccccgaccacatgaagcagcacgacttcttcaagtccgccatgcccgaaggctacgtccaggagcgcaccatcttcttcaaggacgacggcaactacaagacccgcgccgaggtgaagttcgagggcgacaccctggtgaaccgcatcgagctgaagggcatcgacttcaaggaggacggcaacatcctggggcacaagctggagtacaactacaacagccacaacgtctatatcatggccgacaagcagaagaacggcatcaaggtgaacttcaagatccgccacaacatcgaggacggcagcgtgcagctcgccgaccactaccagcagaacacccccatcggcgacggccccgtgctgctgcccgacaaccactacctgagcacccagtccgccctgagcaaagaccccaacgagaagcgcgatcacatggtcctgctggagttcgtgaccgccgccgggatcactctcggcatggacgagctgtacaagaggcctataagatccggcgccaccaacttctccctgctgaagcaggccggcgacgtggaggagaaccccggccccgcccccagatctcttgcatactacctgcaggataccggtataacgcgtataGCTAGCatgggaactagtgactacaagatcaagctgcgggccgccgagagtgaggtggagaagctgcagacgcgcccagagcgggcggtcaccgagcgggagcggctggagatcaagctggaggcctcgcagagcgaactgggcaagtcgaaggccgagctggagaaggccacctgcgaaatgggcaggagcagcgccgactgggagtccaccaagcagaggaccgcccgcctggagctggagaacgagcggctgaaacacgatctggagcgttcgcagaatgtacaaaagttaatgttcgaaacgggcaagatatcgacaacctttggcaggaccacgatgaccacgtcccaggaactggatcgagcccaggagcgggccgacaaggcctcagccgagctgcgacgcacccaggccgagctgagagtcacacagtcggatgcggaaagagcacgcgaggaggcggccgccctgcaggagaagctggagaagagccagggcgaggtgtaccgactcaaggccaagctggagaacgcccagggcgagcaggagagtctgcgccaggagctggagaaggcgcagagcggtgtctctcgcatccacgccgaccgtgatcgggccttctccgaggtggaaaagatcaaggaggagatggagcgcacccaggccacgttgggcaagtcgcagctgcagcacgagaagctgcagaactcgctggacaaggcccagaacgaagtcgatcatctgcaggataagctggacaaggcctgcacggagaaccgccgtctggtgctcgagaaggagaagctcacctacgactacgacaacctgcagtcgcagctggacaaggccttgggccaggcggccaggatgcagaaggagcgcgagactctctctttggacacggatcgcattcgcgagaagctggagaagacgcaggtgcaactgggtcgcatccagaaggagcgggatcaattctccgacgagctggagacgctcaaggagcggtcggaatcggcgcagacccttctcatgaaggccgcccgcgaccgggaggcgatgcaaacggatctggaagttctcaaggagcgctacgagaaatcgcacgccattcagcagaaactccagatggagcgcgacgatgcggtcaccgaagtcgagatcctcaaggagaaactggacaaggcgctgtacgccagccaaaagctgatcgacgagaaggacacctccaacaaggagttcgaaaagatgctggagaagtacgatcgggcccagaacgagatctatcgccttcagtcccgttgcgatacggcagaagcggatagagcccgcttggaggtggaggcggagcgatctggcctagctgccagcaaggctcgcgaggatctgcgtaagctgcaggacgagagcactcggctgcaggaggcctgcgatcgggcggcgctccagttgagccgcgccaaggagtgcgaggacaatgcgcgcagcgaactggagcacagtcgcgatcgcttcgacaagctacagacggacattcggcgtgcccagggcgagaaggagcacttccagtccgagctggagagggtcacctacgaactggagcgcgcacatgccgcccagaccaaggcgagcgccagcgtggaggcggccaaggaggaggcggcacactatgccgtagagcttgagaagatgcgcgaccgctacgagaagagccaggtggagctgcgcaagctgcaggacacggacaccttcgggcgggagacgcgacgcctcaaggaggagaacgagcggctgcgcgagaagctggacaagacgctcatggaactggagaccatacgcggcaaatcgcagtacgagtcggagtcattcgagaagtacaaggacaagtacgagaagatcgagatggaagtgcagaacatggagtcgaaactgcacgagaccagcctgcagctggagctgtcgaagggcgaggtggccaaaatgctggccaatcaggaaaagcagcgatccgagctggaacgggcgcacatcgagcgggagaaggcacgcgacaagcatgagaagctactgaaggaggtcgatcgtttgcgcctgcaacagtcctcggtgagccccggcgatccggtccgagcgtcgacgtcctcctcttccgctctgtccgctggcgagcggcaggagatcgaccgcctgcgggatcgccttgaaaaggcgctgcagtcgcgtgacgccaccgagctggaggccggtcgcttggccaaggaactggagaaggcgcaaatgggtaccagctcgagatatctagacccagctttcttgtacaaagtggtgagctccgccaccatggtgagcaagggcgaggagaataacatggccatcatcaaggagttcatgcgcttcaaggtgcgcatggagggctccgtgaacggccacgagttcgagatcgagggcgagggcgagggccgcccctacgagggcacccagaccgccaagctgaaggtgaccaagggtggccccctgcccttcgcctgggacatcctaacccccaacttcacctacggctccaaggcctacgtgaagcaccccgccgacatccccgactacttgaagctgtccttccccgagggcttcaagtgggagcgcgtgatgaacttcgaggacggcggcgtggtgaccgtgacccaggactcctccctgcaggacggcgagttcatctacaaggtgaagctgcgcggcaccaacttcccctccgacggccccgtaatgcagaagaagaccatgggctgggaggcctcctccgagcggatgtaccccgaggacggcgccctgaagggcgagatcaagatgaggctgaagctgaaggacggcggccactacgacgctgaggtcaagaccacctacaaggccaagaagcccgtgcagctgcccggcgcctacatcgtcggcatcaagttggacatcacctcccacaacgaggactacaccatcgtggaactgtacgaacgcgccgagggccgccactccaccggcggcatggacgagctgtacaagcaccggtccacgtgaTGATAATAACctaggtctgcgatcgcaaaggcgcgccctagaggatctttgtgaaggaaccttacttctgtggtgtgacataattggacaaactacctacagagatttaaagctctaaggtaaatataaaatttttaagtgtataatgtgttaaactactgattctaattgtttgtgtattttagattccaacctatggaactgatgaatgggagcagtggtggaatgcctttaatgaggaaaacctgttttgctcagaagaaatgccatctagtgatgatgaggctactgctgactctcaacattctactcctccaaaaaagaagagaaaggtagaagaccccaaggactttccttcagaattgctaagttttttgagtcatgctgtgtttagtaatagaactcttgcttgctttgctatttacaccacaaaggaaaaagctgcactgctatacaagaaaattatggaaaaatatttgatgtatagtgccttgactagagatcataatcagccataccacatttgtagaggttttacttgctttaaaaaacctcccacacctccccctgaacctgaaacataaaatgaatgcaattgttgttgttaacttgtttattgcagcttataatggttacaaataaagcaatagcatcacaaatttcacaaataaagcatttttttcactgcattctagttgtggtttgtccaaactcatcaatgtatcttatcatgtctggatccactagaaggcctaattctagtatgtatgtaagttaataaaacccattttttggagaatgtagatttaaaaaaacatattttttttttattttttactgcactggacatcattgaacttatctgatcagttttaaatttacttcgatccaagggtatttgaagtaccaggttctttcgattacctctcactcaaaatgacattccactcaaagtcagcgctgtttgcctccttctctgtccacagaaatatcgccgtctctttcgccgctgcgtccgctatctctttcgccaccgtttgtagcgttacctagcgtcaatgtccgccttcagttgcactttgtcagcggtttcgtgacgaagctccaagcggtttacgccatcaattaaacacaaagtgctgtgccaaaactcctctcgcttcttatttttgtttgttttttgagtgattggggtggtgattggttttgggtgggtaagcaggggaaagtgtgaaaaatcccggcaatgggccaagaggatcaggagctattaattcgcggaggcagcaaacacccatctgccgagcatctgaacaatgtgagtagtacatgtgcatacatcttaagttcacttgatctataggaactgcgattgcaacatcaaattgtctgcggcgtgagaactgcgacccacaaaaatcccaaaccgcaatcgcacaaacaaatagtgacacgaaacagattattctggtagctgtgctcgctatataagacaatttttaagatcatatcatgatcaagacatctaaaggcattcattttcgactacattcttttttacaaaaaatataacaaccagatattttaagctgatcctagatgcacaaaaaataaataaaagtataaacctacttcgtaggatacttcgttttgttcggggttagatgagcataacgcttgtagttgatatttgagatcccctatcattgcagggtgacagcggagcggcttcgcagagctgcattaaccagggcttcgggcaggccaaaaactacggcacgctcctgccacccagtccgccggaggactccggttcagggagcggccaactagccgagaacctcacctatgcctggcacaatatggacatctttggggcggtcaatcagccgggctccggatggcggcagctggtcaaccggacacgcggactattctgcaacgagcgacacataccggcgcccaggaaacatttgctcaagaacggtgagtttctattcgcagtcggctgatctgtgtgaaatcttaataaagggtccaattaccaatttgaaactcagtttgcggcgtggcctatccgggcgaacttttggccgtgatgggcagttccggtgccggaaagacgaccctgctgaatgcccttgcctttcgatcgccgcagggcatccaagtatcgccatccgggatgcgactgctcaatggccaacctgtggacgccaaggagatgcaggccaggtgcgcctatgtccagcaggatgacctctttatcggctccctaacggccagggaacacctgattttccaggccatggtgcggatgccacgacatctgacctatcggcagcgagtggcccgcgtggatcaggtgatccaggagctttcgctcagcaaatgtcagcacacgatcatcggtgtgcccggcagggtgaaaggtctgtccggcggagaaaggaagcgtctggcattcgcctccgaggcactaaccgatccgccgcttctgatctgcgatgagcccacctccggactggactcatttaccgcccacagcgtcgtccaggtgctgaagaagctgtcgcagaagggcaagaccgtcatcctgaccattcatcagccgtcttccgagctgtttgagctctttgacaagatccttctgatggccgagggcagggtagctttcttgggcactcccagcgaagccgtcgacttcttttcctagtgagttcgatgtgtttattaagggtatctagcattacattacatctcaactcctatccagcgtgggtgcccagtgtcctaccaactacaatccggcggacttttacgtacaggtgttggccgttgtgcccggacgggagatcgagtcccgtgatcggatcgccaagatatgcgacaattttgctattagcaaagtagcccgggatatggagcagttgttggccaccaaaaatttggagaagccactggagcagccggagaatgggtacacctacaaggccacctggttcatgcagttccgggcggtcctgtggcgatcctggctgtcggtgctcaaggaaccactcctcgtaaaagtgcgacttattcagacaacggtgagtggttccagtggaaacaaatgatataacgcttacaattcttggaaacaaattcgctagattttagttagaattgcctgattccacacccttcttagtttttttcaatgagatgtatagtttatagttttgcagaaaataaataaatttcatttaactcgcgaacatgttgaagatatgaatattaatgagatgcgagtaacattttaatttgcagatggttgccatcttgattggcctcatctttttgggccaacaactcacgcaagtgggcgtgatgaatatcaacggagccatcttcctcttcctgaccaacatgacctttcaaaacgtctttgccacgataaatgtaagtcttgtttagaatacatttgcatattaataatttactaactttctaatgaatcgattcgatttaggtgttcacctcagagctgccagtttttatgagggaggcccgaagtcgactttatcgctgtgacacatactttctgggcaaaacgattgccgaattaccgctttttctcacagtgccactggtcttcacggcgattgcctatccgatgatcggactgcgggccggagtgctgcacttcttcaactgcctggcgctggtcactctggtggccaatgtgtcaacgtccttcggatatctaatatcctgcgccagctcctcgacctcgatggcgctgtctgtgggtccgccggttatcataccattcctgctctttggcggcttcttcttgaactcgggctcggtgccagtatacctcaaatggttgtcgtacctctcatggttccgttacgccaacgagggtctgctgattaaccaatgggcggacgtggagccgggcgaaattagctgcacatcgtcgaacaccacgtgccccagttcgggcaaggtcatcctggagacgcttaacttctccgccgccgatctgccgctggactacgtgggtctggccattctcatcgtgagcttccgggtgctcgcatatctggctctaagacttcgggcccgacgcaaggagtagccgacatatatccgaaataactgcttgtttttttttttaccattattaccatcgtgtttactgtttattgccccctcaaaaagctaatgtaattatatttgtgccaataaaaacaagatatgacctatagaatacaagtatttccccttcgaacatccccacaagtagactttggatttgtcttctaaccaaaagacttacacacctgcataccttacatcaaaaactcgtttatcgctacataaaacaccgggatatattttttatatacatacttttcaaatcgcgcgccctcttcataattcacctccaccacaccacgtttcgtagttgctctttcgctgtctcccacccgctctccgcaacacattcaccttttgttcgacgaccttggagcgactgtcgttagttccgcgcgattcggttcgctcaaatggttccgagtggttcatttcgtctcaatagaaattagtaataaatatttgtatgtacaatttatttgctccaatatatttgtatatatttccctcacagctatatttattctaatttaatattatgactttttaaggtaattttttgtgacctgttcggagtgattagcgttacaatttgaactgaaagtgacatccagtgtttgttccttgtgtagatgcatctcaaaaaaatggtgggcataatagtgttgtttatatatatcaaaaataacaactataataataagaatacatttaatttagaaaatgcttggatttcactggaactagaattaattcggctgctgctctaaacgacgcatttcgtactccaaagtacgaattttttccctcaagctcttattttcattaaacaatgaacaggacctaacgcacagtcacgttattgtttacataaatgattttttttactattcaaacttactctgtttgtgtactcccactggtatagccttcttttatcttttctggttcaggctctatcactttactaggtacggcatctgcgttgagtcgcctccttttaaatgtctgaccttttgcaggtgcagccttccactgcgaatcattaaagtgggtatcacaaatttgggagttttcaccaaggctgcacccaaggctctgctcccacaattttctcttaatagcacacttcggcacgtgaattaattttactccagtcacagctttgcagcaaaatttgcaatatttcatttttttttattccacgtaagggttaatgttttcaaaaaaaaattcgtccgcacacaacctttcctctcaacaagcaaacgtgcactgaatttaagtgtatacttcggtaagcttcggctatcgacgggaccaccttatgttatttcatcatgggccagacccacgtagtccagcggcagatcggcggcggagaagttaagcgtctccaggatgaccttgcccgaactggggcacgtggtgttcgacgatgtgcagctaatttcgcccggctccacgtccgcccattggttaatcagcagaccctcgttggcgtaacggaaccatgagaggtacgacaaccatttgaggtatactggcaccgagcccgagttcaagaagaaggcgtttttccataggctccgcccccctgacgagcatcacaaaaatcgacgctcaagtcagaggtggcgaaacccgacaggactataaagataccaggcgtttccccctggaagctccctcgtgcgctctcctgttccgaccctgccgcttaccggatacctgtccgcctttctcccttcgggaagcgtggcgctttctcaatgctcacgctgtaggtatctcagttcggtgtaggtcgttcgctccaagctgggctgtgtgcacgaaccccccgttcagcccgaccgctgcgccttatccggtaactatcgtcttgagtccaacccggtaagacacgacttatcgccactggcagcagccactggtaacaggattagcagagcgaggtatgtaggcggtgctacagagttcttgaagtggtggcctaactacggctacactagaaggacagtatttggtatctgcgctctgctgaagccagttaccttcggaaaaagagttggtagctcttgatccggcaaacaaaccaccgctggtagcggtggtttttttgtttgcaagcagcagattacgcgcagaaaaaaaggatctcaagaagatcctttgatcttttctacggggtctgacgctcagtggaacgaaaactcacgttaagggattttggtcatgagattatcaaaaaggatcttcacctagatccttttaaattaaaaatgaagttttaaatcaatctaaagtatatatgagtaaacttggtctgacagttaccaatgcttaatcagtgaggcacctatctcagcgatctgtctatttcgttcatccatagttgcctgactccccgtcgtgtagataactacgatacgggagggcttaccatctggccccagtgctgcaatgataccgcgagacccacgctcaccggctccagatttatcagcaataaaccagccagccggaagggccgagcgcagaagtggtcctgcaactttatccgcctccatccagtctattaattgttgccgggaagctagagtaagtagttcgccagttaatagtttgcgcaacgttgttgccattgctacaggcatcgtggtgtcacgctcgtcgtttggtatggcttcattcagctccggttcccaacgatcaaggcgagttacatgatcccccatgttgtgcaaaaaagcggttagctccttcggtcctccgatcgttgtcagaagtaagttggccgcagtgttatcactcatggttatggcagcactgcataattctcttactgtcatgccatccgtaagatgcttttctgtgactggtgagtactcaaccaagtcattctgagaatagtgtatgcggcgaccgagttgctcttgcccggcgtcaacacgggataataccgcgccacatagcagaactttaaaagtgctcatcattggaaaacgttcttcggggcgaaaactctcaaggatcttaccgctgttgagatccagttcgatgtaacccactcgtgcacccaactgatcttcagcatcttttactttcaccagcgtttctgggtgagcaaaaacaggaaggcaaaatgccgcaaaaaagggaataagggcgacacggaaatgttgaatactcatactcttcctttttcaatattattgaagcatttatcagggttattgtctcatgagcggatacatatttgaatgtatttagaaaaataaacaaataggggttccgcgcacatttccccgaaaagtgccacctgacgtctaagaaaccattattatcatgacattaacctataaaaataggcgtatcacgaggccctttcgtctcgcgcgtttcggtgatgacggtgaaaacctctgacacatgcagctcccggagacggtcacagcttgtctgtaagcggatgccgggagcagacaagcccgtcagggcgcgtcagcgggtgttggcgggtgtcggggctggcttaactatgcggcatcagagcagattgtactgagagtgcaccatatggtcgacgatgtaggtcacggtctcgaagccgcggtgcgggtgccagggcgtgcccttgggctccccgggcgcgtactccacctcacccatctggtccatcatgatgaacgggtcgaggtggcggtagttgatcccggcgaacgcgcggcgcaccgggaagccctcgccctcgaaaccgctgggcgcggtggtcacggtgagcacgggacgtgcgacggcgtcggcgggtgcggatacgcggggcagcgtcagcgggttctcgacggtcacggcgggcatgtcgaccatatgcggtgtgaaataccgcaccgaatcgcgcggaactaacgacagtcgctccaaggtcgtcgaacaaaaggtgaatgtgttgcggagagcgggtgggagacagcgaaagagcaactacgaaacgtggtgtggtggaggtgaattatgaagagggcgcgcgatttgaaaagtatgtatataaaaaatatatcccggtgttttatgtagcgataaacgagtttttgatgtaaggtatgcaggtgtgtaagtcttttggttagaagacaaatccaaagtctacttgtggggatgttcgaaggggaaatacttgtattctataggtcatatcttgtttttattggcacaaatataattacattagctttttgagggggcaataaacagtaaacacgatggtaataatggtaaaaaaaaaaacaagcagttatttcggatatatgtcggctactccttgcgtcgggcccgaagtcttagagccagatatgcgagcacccggaagctcacgatgagaatggccagaccatgatgaaataacataaggtggtcccgtcggcaagagacatccacttaacgtatgcttgcaataagtgcgagtgaaaggaatagtattctgagtgtcgtattgagtctgagtgagacagcgatatgattgttgattaacccttagcatgtccgtggggtttgaattaactcataatattaattagacgaaattatttttaaagttttatttttaataatttgcgagtacgcaaagcttggctgcatccaacgcgttgggagctctccggatccaagcttgcatgcctgcaggt
